# Enhancing the endocannabinoid system to treat residual disease in relapse-free multiple sclerosis

**DOI:** 10.3389/fneur.2026.1747131

**Published:** 2026-03-04

**Authors:** Pietro Annovazzi, Marinella Clerico, Eleonora Cocco, Antonella Conte, Girolama Alessandra Marfia, Marco Salvetti, Valentina Tomassini, Rocco Totaro, Diego Centonze

**Affiliations:** 1 Neuroimmunology Unit, ASST Valle Olona, Gallarate Hospital, Gallarate, VA, Italy; 2 Clinical and Biological Sciences Department, University of Torino, Torino, Italy; 3 Medical Sciences and Public Health Department, University of Cagliari, Cagliari, Italy; 4 Multiple Sclerosis Center, Cagliari ASL, Cagliari, Italy; 5 Department of Human Neurosciences, Sapienza University, Rome, Italy; 6 Unit of Neurology, IRCCS Neuromed, Pozzilli, IS, Italy; 7 Department of Systems Medicine, Tor Vergata University, Rome, Italy; 8 Department of Neurosciences, Mental Health and Sensory Organs, Sapienza University, Rome, Italy; 9 Department of Neurosciences, Imaging and Clinical Sciences, University G. D’Annunzio, Chieti-Pescara, Italy; 10 Demyelinating Disease Center Department of Neurology, San Salvatore Hospital, L’Aquila, Italy

**Keywords:** algorithms of treatment, lifestyle interventions, nabiximols, Smouldering Disease, spasticity-plus-syndrome

## Abstract

The recent introduction of High-Efficacy Therapies (HETs) in clinical practice has drastically reduced the frequency of acute inflammatory episodes and relapses, in patients with Multiple Sclerosis (MS), gradually shifting the interest of clinicians toward preventing disease progression and treating symptoms associated with the residual disease. This article summarizes the output of a recent meeting (June 2025, in Rome) among an Italian group of neurologists, who discussed about published evidence supporting the involvement of the endocannabinoid system (ECS) in MS spasticity and its associated symptoms. Sharing their clinical experiences about the silent progression of the disease, in patients with Relapse-Free Multiple Sclerosis (RFMS), treated with HETs, authors propose a new algorithm to treat residual disease in RFMS, by enhancing ECS with both cannabinoid agents and lifestyle interventions (diet and physical activity).

## 1 Introduction

Multiple Sclerosis (MS) is the most common chronic autoimmune neurodegenerative disease in young Caucasian adults (1). It is characterized by lesions of oligodendrocytes and myelin, in addition to neuronal and axonal injury, resulting in multiple neurological dysfunctions (1).

One of the clinical features for many MS patients is relapses, that are episodes of neurological worsening that evolve over hours or days and then last for days or weeks, followed by varying degrees of recovery (2). MS relapses are typically accompanied by changes in magnetic resonance imaging (MRI) and contribute to meaningful neurological disability over the short term (2). Most patients (85–90%) present with Relapsing–Remitting MS (RRMS) at disease onset (3).

With earlier diagnosis of MS and hence earlier treatment with High-Efficacy Therapies (HETs), such as Disease Modifying Treatments (DMTs), the number and the severity of relapses can be reduced (3). Nowadays, early intervention with DMTs improves short-term and long-term clinical outcomes in MS patients, but disability worsening may also occur in the absence of relapses or new MRI lesions (3). This is often referred to as Progression Independent of Relapse and MRI Activity (PIRMA) or Smouldering Disease (SD) (4).

A range of evidence has shown that the ‘real MS’ is driven primarily by a smouldering pathological disease process (4). Relapses and focal activity revealed by MRI, in MS patients on placebo or on DMTs, were found to be poor predictors of long-term disease evolution and were dissociated from disability outcomes. In addition, the progressive accumulation of disability can occur independently of relapses, from early in the disease course (4). This ‘silent progression’ of the disease, in RRMS patients, underlies a neurodegenerative process, with both focal and diffuse tissue destructive components, and with inflammation and neurodegeneration occurring throughout the disease spectrum (2).

Patients with SD can develop symptoms in any number of domains across mobility, spasticity, pain, hand function, vision, fatigue, cognition, bowel/bladder function, sensory function, depression and tremor/coordination, with considerable inter-individual variation in the patterns of impairment (5). Worsening spasticity can negatively impact other concomitant SD symptoms, which makes its management particularly relevant, because of its great potential to relieve the cluster of inter-related symptoms (6).

## 2 The concept of spasticity-plus-syndrome

The traditional approach to management of SD-related symptoms has been “organ-oriented,” aiming to resolve complications at a local level (7). Many different molecules are generally used as symptomatic agents, alone or in combination: anticonvulsants, antispasmodics and antidepressants, all aiming at targeting one specific symptom (8). This approach inevitably implies an increased risk of side effects, with a potential worsening of the symptoms and reduced patients’ compliance with prescribed treatments (8).

**Figure 1:**
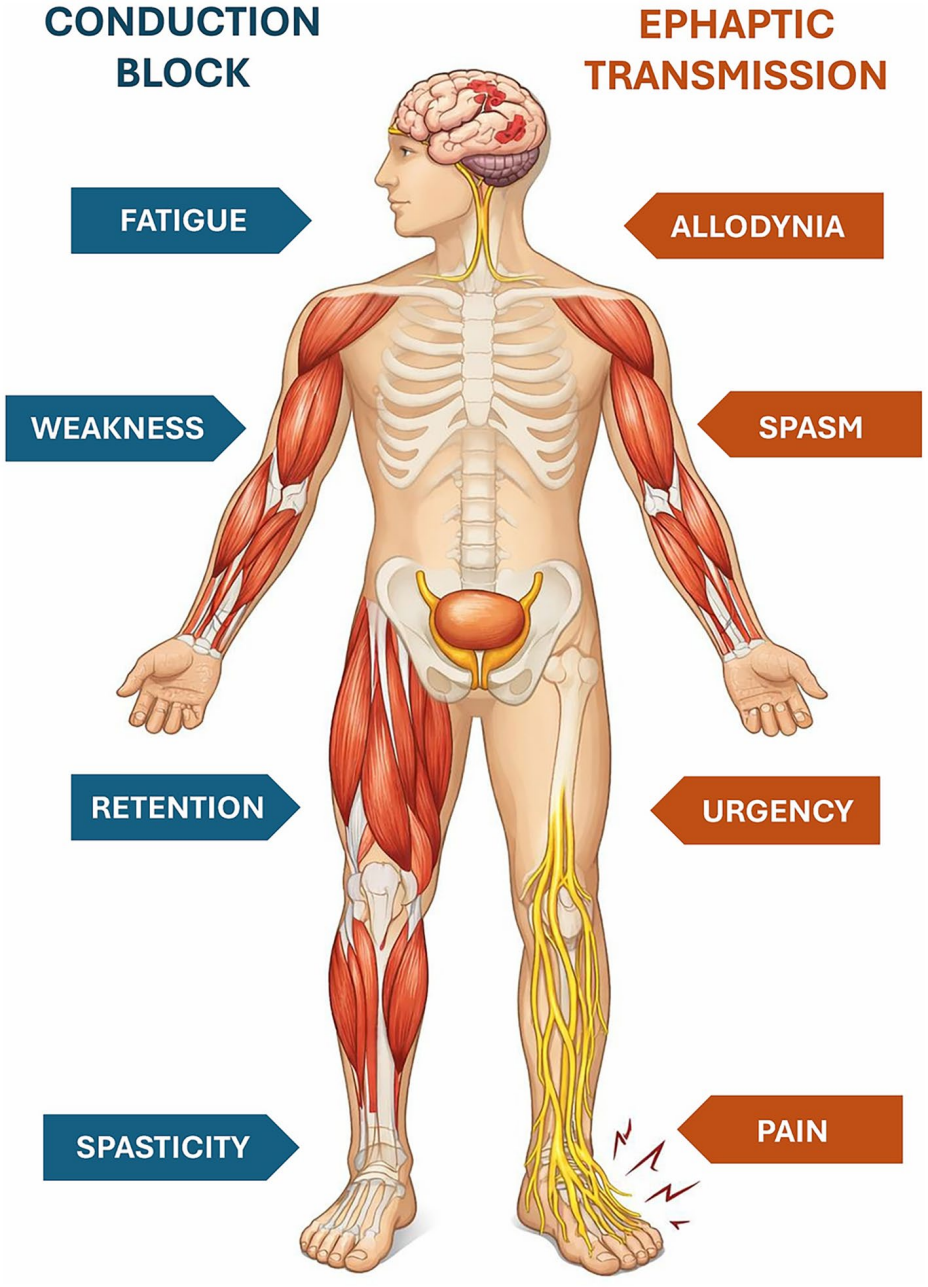
The spasticity-plus syndrome (SPS) model characteristics. The spasticity-plus syndrome model supports the hypothesis that spasticity, triggered by central nervous system (CNS) damage, and other symptoms, caused by axon demyelination in terms of conduction block (spasticity, fatigue, weakness, and retention) and ephaptic transmission (spasms, pain, allodynia, and urgency) are placed on the same level. Modified from Bruno et al. (8).

The spasticity-plus syndrome (SPS) was first introduced by Fernández et al. (9), who defined spasticity and other accompanying symptoms as a cluster of clinical manifestations independently linked by a common underlying pathophysiology (10). The novelty of this approach is that it allows putting spasticity and other symptoms (pain, allodynia, bladder dysfunction, fatigue, and sleep disturbances) on the same level (8). Recognition of SPS as a distinct clinical entity implies that a single drug interfering with its underlying pathophysiology may be able to act simultaneously on all the symptoms that are part of it. It also implies that a given treatment can differently affect the various manifestations of the syndrome in a variable manner, depending on the patient’s specific characteristics and pharmacological responsiveness (Figure 1) (8).

The SPS model can simplify the approach to symptomatic management of SD patients in daily practice, potentially reducing the risks associated with polypharmacy (7).

## 3 Role of cannabinoids in MS

The dysregulation of endocannabinoid system (ECS) in MS has been extensively documented by clinical and preclinical literature (10).

Cannabinoid receptors 1 (CBR1) and CBR2 are abundant in the CNS, with a higher concentration in the brainstem (1). CBR1 is mainly localized at the presynaptic terminal and is involved in synaptic modulation by inhibiting the release of other neurotransmitters. The distribution of CBR2 in the CNS is concentrated mainly along the sensory pathway, making this receptor a target for neuropathic pain therapy. Moreover, CBR2 is also localized on microglia and plays a role in immune modulation and neuronal microglial interaction (8).

Evidence suggests that cannabinoids may interact with axonal channels reducing their excitotoxic potential in several pathological conditions (8). In MS, voltage-gated channels are reduced, and action potential propagation is compromised (1). Neurons activate compensatory mechanisms such as an ectopic expression of sodium voltage-dependent channels, which can lead to axonal damage in the long-term (1). Preclinical studies showed that cannabinoids reduce the hyperexcitability of sodium voltage-dependent channels and diminish the excitotoxic sodium and calcium currents. Hence, cannabinoids can act on these channels to relieve MS symptoms (1).

Preclinical studies on MS murine models demonstrated that cannabinoids counteract the neurodegenerative process that leads to chronic disability in the experimental autoimmune encephalomyelitis (EAE), reducing excitotoxicity and oxidative stress and promoting neurogenesis. Moreover, cannabinoids inhibit the breakdown of myelin, preventing or reversing the demyelination process (10). Anandamide significantly attenuates neuroinflammation, by inhibiting microglial activation and reducing the release of interleukin (IL)-23 and IL-12 (10). Moreover, cannabidiol decreased inflammation, microglia activation, and T-lymphocyte recruitment in the spinal cord (10).

ECS influences synaptic plasticity, brain atrophy, and risk of progression in MS (10). Considering that both PIRMA and SPS symptoms can develop early in the disease course, early treatment with drugs interacting with ECS may represent a breakthrough in the treatment of SD symptoms (10).

## 4 Nabiximols: efficacy of an ECS modulator in SD symptoms

In the early 2000s, two randomized controlled trials showed that two compounds extracted from the *Cannabis sativa*, delta-9-tetrahydrocannabinol (THC) and cannabidiol (CBD), improve gait control, balance, spasm frequency, and insomnia (10).

Nabiximols, an oromucosal spray that comprises a balanced 1:1 ratio mixture of THC and CBD, is approved as a second-line option for symptomatic management of MS spasticity and has been available in Europe for more than a decade (10, 11). It is indicated as add-on treatment for symptom improvement in adult patients with moderate-to-severe MS spasticity who have not responded adequately to other medications and who demonstrated clinically significant improvement in spasticity-related symptoms during an initial trial of therapy (11).

It acts as a modulator of the human ECS through interaction with CBR1 and CBR2. As a partial agonist of CBR1, THC may modulate the effects of excitatory (glutamic acid) and inhibitory (GABA) neurotransmitters, resulting in muscle relaxation and symptomatic improvement of spasticity. CBD, in contrast, has little affinity for CBR1 and CBR2 and acts as an antagonist at CBR1. THC and CBD are thought to work synergistically, such that CBD counteracts some of the undesirable effects of THC (e.g., sedation, anxiety), while enhancing its beneficial clinical effects, such as analgesia and neuroprotection (11).

Between 2000 and 2010, nabiximols underwent a large clinical trials program involving more than 2,500 MS patients, of whom more than 660 were treated continuously for at least 6 months, yielding more than 1,200 patient-years of clinical experience. Vast clinical experience with nabiximols in the post-marketing setting accounts for more than 150,000 patient-years to date, which is important about monitoring of adverse drug reactions and gathering real-world evidence about its effectiveness (11).

### 4.1 Efficacy on spasticity (before SPS)

**Figure 2:**
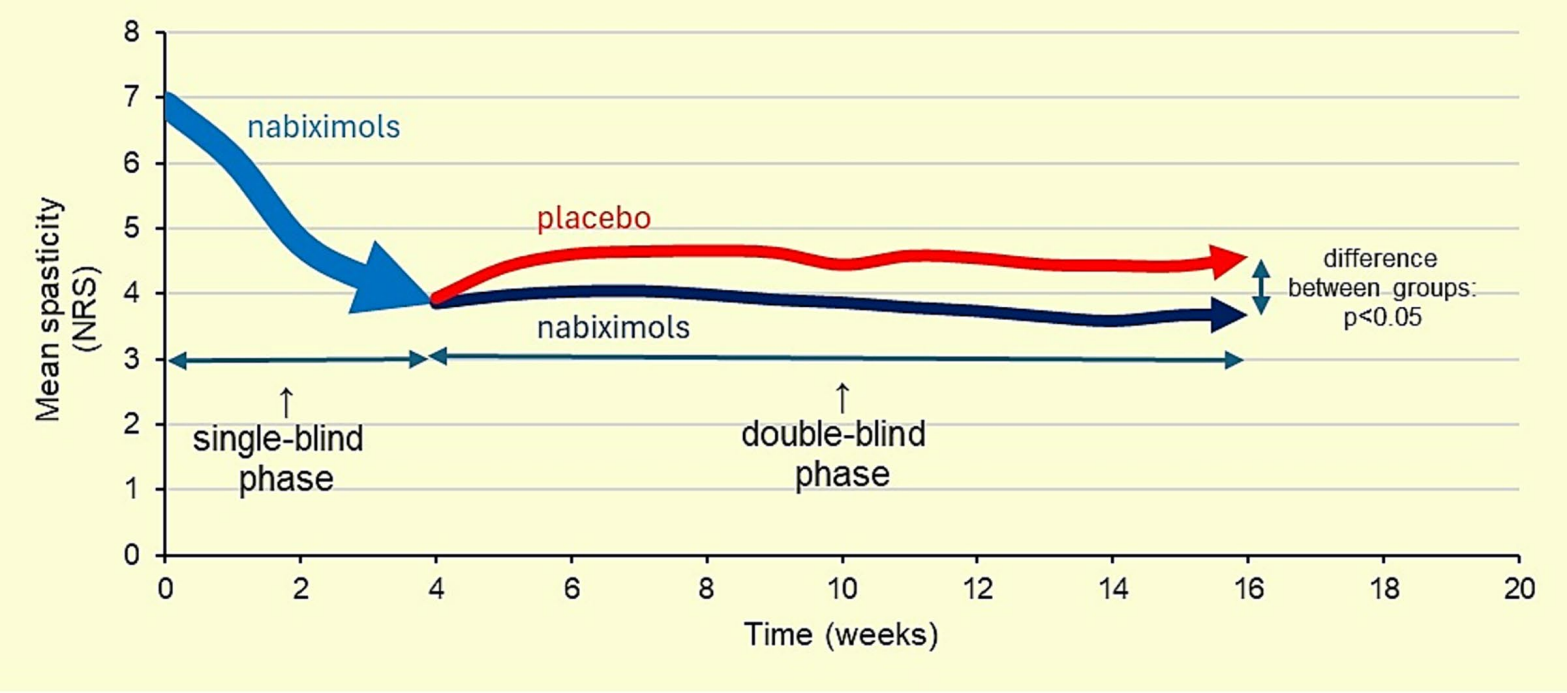
Numeric rating scale (NRS) spasticity scores during a 19-week study in patients with multiple sclerosis (MS) spasticity (intention-to-treat analysis). Subjects were treated with nabiximols, as add-on therapy, in a single-blind manner for 4 weeks, after which those achieving an improvement in spasticity of ≥20% progressed to a 12-week randomized, placebo-controlled phase. Of the 572 subjects enrolled, 272 achieved a ≥20% improvement after 4 weeks of single-blind treatment, and 241 were randomized. The mean change in spasticity 0–10 NRS score at the end of the 4-week single-blind treatment with nabiximols was a decrease (improvement) of 3.01 (±standard deviation, SD = 1.38) points. Over the course of the 12-week randomized phase, the mean spasticity score had further improved in the active treatment group by 0.04 units, from a baseline score of 3.87 points. Adapted with permission from Novotna et al. (12).

Earlier studies showed a significant improvement in the patient-reported severity of spasticity, in patients with MS treated with nabiximols, using a validated 0–10 Numerical Rating Scale (NRS) (Figure 2) (12, 13).

Several randomized controlled trials exploring the efficacy and safety of nabiximols have reported a significant reduction in spasticity compared with placebo, a rapid occurrence of treatment response (within the first 4 weeks), a persistent efficacy over time without need to increase the dosage, and a low drug discontinuation due to side effects or inefficacy (10%) (12–15).

Furthermore, the effectiveness and safety of nabiximols in everyday clinical practice were confirmed by the Mobility Improvement (MOVE) 2 study, a prospective, 3-month non-interventional study. It was extended to 12 months (MOVE 2 prolongation study), confirming the long-term effectiveness and tolerability of nabiximols for the treatment of resistant MS (16, 17).

In the post-approval setting over the 10-year period 2011–2021, numerous real-world evidence studies have provided short- and long-term data about the effectiveness of nabiximols in patients with MS spasticity treated in the routine outpatient setting according to the approved label (11).

### 4.2 Efficacy on spasticity related symptoms (after SPS)

Two AIFA (Agenzia Italiana del Farmaco) e-Registry analyses have investigated the evolution of predefined MS spasticity related symptoms (spasms/cramps, clonic movements, sleep disturbances, urinary dysfunction, pain, depressed mood, trigeminal neuralgia) in patients with moderate-to-severe MS spasticity receiving nabiximols in daily clinical practice (7, 18).

**Figure 3:**
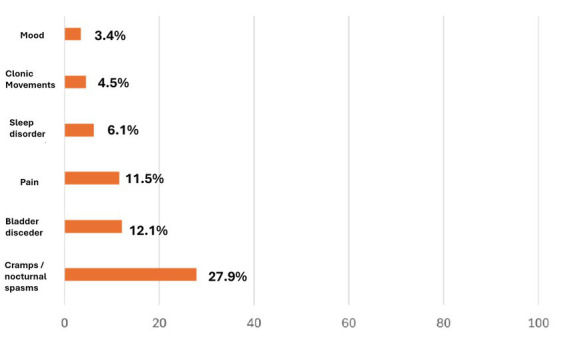
Spasticity-related symptoms reported as improved in the study population. Multiple sclerosis (MS) patients with drug-resistant spasticity were recruited from 30 Italian MS centers. Out of 1,615 enrolled patients, 1,432 reached the end of the first month trial period (T1). Of these, 1,010 patients (70.5%) reached a ≥20% numeric rating scale (NRS) score reduction compared with baseline. Cramps/nocturnal spasms were reported as meaningfully ameliorated in 27.9% of patients, followed by bladder disorders (12.1%), and pain (11.5%). Adapted with permission from Patti et al. (18).

In the first analysis, a relevant proportion (43.8%) of MS patients, with moderate to severe treatment-resistant spasticity, reported meaningful symptomatic relief (spasticity-related symptom - SRS -subgroup) with nabiximols, improving bladder control, sleep quality, pain, and/or mood disorders. About 80% of these SRS responders reported an amelioration in at least 2 spasticity-related symptoms (18). The analysis focused on patients who reported an improvement in spasticity-related symptoms but not reaching the ≥20% NRS score reduction and, thus, considered to be non-responders according to the AIFA requirements. Interestingly, 19.9% of the patients considered non-responders to nabiximols reported a meaningful improvement in one or more spasticity-related symptoms (Figure 3) (18). These findings suggest that the therapeutic benefit of nabiximols may extend beyond muscle tone control (11).

The second and more recent analysis, involving 1,138 patients, confirmed the resolution of MS spasticity-associated symptoms over the longer term (18 months) in treatment continuers (7).

The mean number of MS spasticity-associated symptoms present at baseline decreased by 33% at Week 4 and by 29% at 18 months in treatment continuers. Pain, the most common associated symptom at baseline, was frequently clustered with sleep disturbances and spasms/cramps, supporting the concept of a spasticity-plus syndrome. At 18 months, symptom resolution rates in treatment continuers were ≥50% for all associated symptoms, including pain (7).

Although the mean number of spasticity-associated symptoms was reduced to a greater extent from baseline to 18 months, in patients with ≥20% or ≥30% NRS response, a reduction was also observed in NRS non-responders (7). Factors associated with an increased probability of continuing nabiximols treatment, such as higher baseline NRS scores and a greater initial NRS response, emphasize the importance of early monitoring of patients to identify those most likely to benefit (7).

A recent multicentric, observational, real-life study confirms nabiximols as an effective, safe and well-tolerated treatment option for resistant MS spasticity and spasticity-related symptoms (pain, bladder dysfunction and gait) (19).

Looking ahead, confirmation of the existence of SPS and the role of nabiximols in managing the syndrome may have important implications for optimizing symptom treatment in MS patients (2, 20). Diagnosis of SPS can simplify pharmaceutical treatment of symptoms in MS, which would help to avoid or reduce side effects and drug interactions associated with polypharmacy (21).

Evidence suggests that an assessment of clinical benefit with nabiximols may need to consider evolution in related symptoms, not spasticity alone. Using appropriate assessment tools, patients who show improvement or resolution of associated symptoms might be candidates for continued nabiximols treatment, irrespective of their spasticity improvement status, based on current NRS thresholds. This is an intriguing concept, as it may extend the opportunity for benefit with nabiximols to a wider group of patients with MS spasticity (11).

Moreno-Martet et al. proposed an alternative murine model of MS, the experimental autoimmune encephalomyelitis (EAE), which provides a useful model to reproduce MS progression and the underlying pathological mechanisms. The authors showed that administering nabiximols-like drugs intraperitoneally at the onset of symptoms and continuing until the first relapse of the disease could mitigate the progression of neurological deficits. In this respect, nabiximols mechanism of action is not only mediated by an interaction with CBR1 and CBR2 in neurons of the frontal and prefrontal motor cortex but also involves interference with MS-specific demyelination and axonal pathology (22).

### 4.3 Safety profile of nabiximols

In real-life studies, patients treated with nabiximols showed high treatment satisfaction and tolerability scores (17, 19). Across studies, treatment-related adverse events (AEs) occurring more frequently with nabiximols (*n* = 805) than with placebo (*n* = 741) included dizziness (24.8 vs. 7%), fatigue (11.1 vs. 6.6%) and somnolence (8.1 vs. 1.9%). These events tended to occur during the first 4 weeks of exposure and were usually mild to moderate in severity and resolved quickly (11). Pharmacovigilance data accumulated during short- and long-term use of nabiximols in daily practice, with the approved posology, supports the safety profile reported in randomized controlled trials (11).

## 5 Nabiximols and lifestyle interventions

Multisymptomatic chronic diseases, such as MS, require a holistic management approach. To treat chronic diseases effectively, it is essential to understand the impact of the disease (and individual symptoms)—as well as the impact of an intervention—on a patient’s life (23). For all patients, it is useful to encourage attention to a healthy lifestyle, including maintaining an optimistic outlook, a healthy diet, and regular exercise as tolerated (24).

Despite a normal calorie intake, patients with MS often have an imbalance between macronutrients intake with low-carbohydrate and high-lipid diet associated with abdominal obesity, higher body mass index (BMI), waist-to-hip ratio, waist-to-height ratio, and higher fat percentage (25).

A high-fat diet significantly alters the gut microbiota and modulates the endocannabinoidome (eCBome), increasing CBR1 and triglyceride accumulation, while decreasing gut microbiota diversity (26). Compositional changes to the gut microbiome triggered by dietary interventions correlate with differential expressions of ECS components and altered profiles of bioactive lipids in the blood stream and intestinal tissues (27). These findings suggest a reciprocal relationship between the host ECS and the gut microbiome. More recently, experimental evidence has emerged demonstrating that endocannabinoids and their congeners can modulate bacterial functions, physiology, and behaviors (27).

While exercise was a controversial treatment as it was thought to exacerbate the symptoms of MS, today, physical activity is considered not only safe without serious adverse effects, but also beneficial on disease symptoms. Healthcare providers should encourage ≥150 min/week of physical activity, and early evaluation by a specialist is recommended to ensure individualized treatment (25).

For decades, it was hypothesized that exercise-induced endorphin release is solely responsible for a runner’s high, but recent evidence has suggested that endocannabinoids may also play a role, particularly in anxiolysis and pain reduction (28).

Given the very important role that the ECS plays in diet and exercise, neurologists should also recommend substantial lifestyle changes, to potentially reduce silent progression during SD.

## 6 New algorithm for the treatment of SD

Evidence selected for this review have been discussed in a recent meeting among an Italian group of neurologists, who introduced published data and their personal clinical experiences, regarding the potential involvement of ECS, in MS spasticity and its associated symptoms. The workshop was held in Rome, and it was divided into two parts: the first one in April 2025 and the second in June 2025.

The first result of the meeting was the proposition that SD may be treated by enhancing ECS as early as possible. To this purpose, both cannabinoid agents and lifestyle interventions (diet and physical activity) can be exploited. As result of the discussion, authors proposed a treatment algorithm that considers the involvement of ECS in the silent progression of the residual disease, in RFSM patients.

**Figure 4:**
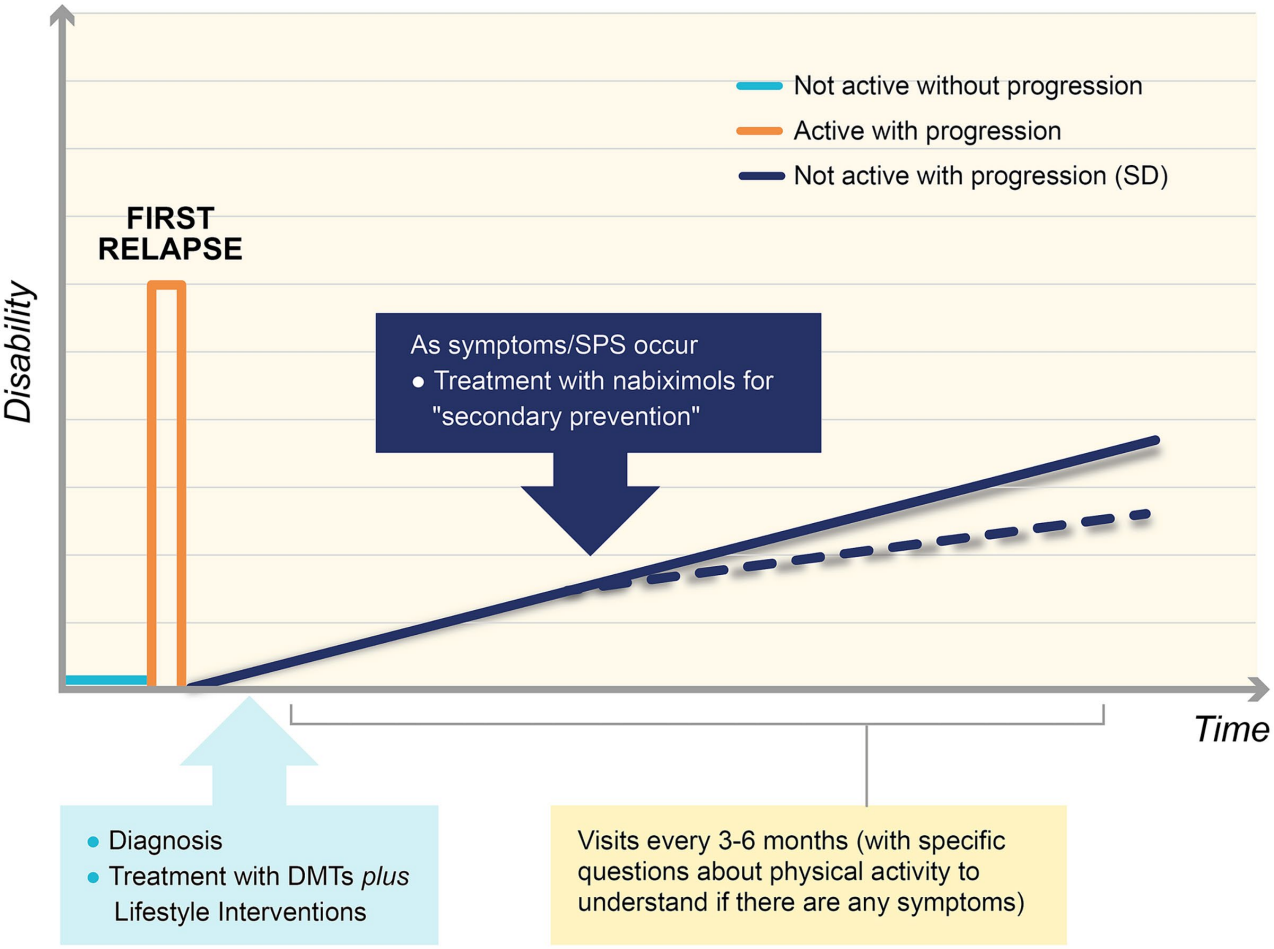
New algorithm for the treatment of Smouldering Disease (SD). Multiple sclerosis (MS) is diagnosed with the first relapse of the disease (orange line). Patients experience spasticity and are treated with disease modifying therapies (DMTs) to prevent further relapses. Combining DMTs and lifestyle interventions can slow down the progression of disease, decreasing disability (blue continuous line). If any symptoms in the spasticity-plus syndrome (SPS) cluster appear, it is advisable that neurologists begin treatment with an endocannabinoid agent, such as nabiximols, as secondary prevention, to lower the slope of the symptomatic worsening curve (blue dotted line).

Currently, MS is diagnosed with the first relapse of the disease. Patients experience spasticity and are treated with DMTs to prevent further relapses. In this way, they can experience no symptoms for a certain period. Combining advanced therapy with lifestyle interventions would help stimulate the ECS and so slow the progression of SD (Figure 4).

The authors believe that it is extremely important to monitor patients with visits every 3–6 months, including a careful assessment of physical function (“stress test”). If any symptoms in the SPS cluster appear, it is advisable that MS neurologists early begin treatment with an endocannabinoid agent, such as nabiximols, as secondary prevention, to lower the slope of the symptomatic worsening curve, so slowing the silent progression of residual disease (Figure 4).

Periodical practice of the “stress test” is a useful tool, able to detect even the slightest symptoms and to intervene early with an endocannabinoid. The correct way to monitor a patient’s adherence to physical activity or diet allows clinicians to know when a patient is starting to experience difficulties, as early as possible. Getting patients to do physical activity through standardized guidelines is a bit complicated, except for those patients who are already active or have practically no neurological impairment whatsoever, which is a very small segment of patients. For this reason, standardized or remote programs may be useful.

**Figure 5:**
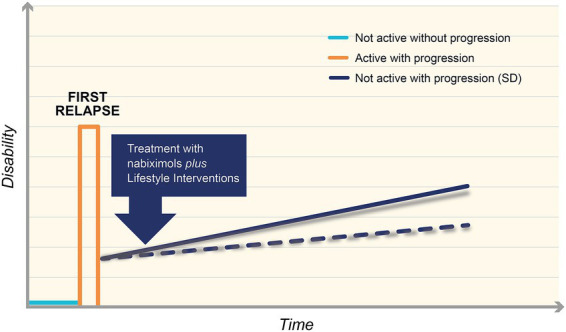
New algorithm for the treatment of Smouldering Disease (SD) in patients with Relapse-Associated Worsening (RAW). In case of RAW, after the first relapse (orange line), patients do not return to baseline wellbeing, but they have already worsened disability, with symptoms at rest: early intervention with an endocannabinoid agent, such as nabiximols, combined with diet and physical activity, can be effective. Blue continuous line represents the silent progression of disease; blue dotted line represents the potential slope’s reduction of the symptomatic worsening curve, following the early intervention on endocannabinoid system.

Again, based on poor ECS stimulation in SD, authors presented a similar algorithm specific for patients with Relapse-Associated Worsening (RAW). In this case, after the first relapse, patients do not return to baseline wellbeing, but they have already worsened disability, with symptoms at rest: early intervention with an endocannabinoid agent, such as nabiximols, combined with diet and physical activity, can be effective (Figure 5).

Similarly, in PIRMA, early treatment with nabiximols *plus* lifestyle interventions may be effective to reduce disease progression and to improve patient wellbeing.

Authors acknowledge that this literature review is the result of their discussion and shared clinical experiences. The presented algorithms offer a starting point for better monitoring silent disease progression in patients with multiple sclerosis.

## 7 Conclusion

Nowadays, MS is effectively controlled thanks to HETs, but monitoring patients is crucial to prevent disease progression and to treat symptoms associated with the residual disease. The smouldering pathological disease process is associated with a dysregulation of ECS, and it underlies a neurodegenerative process, with a silent progressive accumulation of disability. Neurologists should combine DMTs with substantial lifestyle changes, since both diet and physical activity are associated with an enhancement of ECS.

Nabiximols is the first cannabinoid-based drug, approved in 2010, as an add-on treatment for symptoms improvement in adult patients with moderate-to-severe MS spasticity (2). For 10 years real-world use of nabiximols, no new safety signals or evidence of abuse, tolerance or dependence potential have emerged (2). Official guidelines recognize its role in the symptomatic management of MS spasticity (2).

With the aim of increasing clinicians’ awareness of the involvement of ECS in the silent progression of the disease in Relapse-Free Multiple Sclerosis (RFMS) patients, authors developed a treatment algorithm, emphasizing the importance of timely intervention both with an increase in endogenous cannabinoids, through diet and physical activity, and with the use of an exogenous cannabinoid agent such as nabiximols.

Further supporting studies will be designed to demonstrate the validity of this therapeutic approach.
