# Correction: Enhancing the endocannabinoid system to treat residual disease in relapse-free multiple sclerosis

**DOI:** 10.3389/fneur.2026.1829952

**Published:** 2026-04-28

**Authors:** Pietro Annovazzi, Marinella Clerico, Eleonora Cocco, Antonella Conte, Girolama Alessandra Marfia, Marco Salvetti, Valentina Tomassini, Rocco Totaro, Diego Centonze

**Affiliations:** 1 Neuroimmunology Unit, ASST Valle Olona, Gallarate Hospital, Gallarate, VA, Italy; 2 Clinical and Biological Sciences Department, University of Torino, Torino, Italy; 3 Medical Sciences and Public Health Department, University of Cagliari, Cagliari, Italy; 4 Multiple Sclerosis Center, Cagliari ASL, Cagliari, Italy; 5 Department of Human Neurosciences, Sapienza University, Rome, Italy; 6 Unit of Neurology, IRCCS Neuromed, Pozzilli, IS, Italy; 7 Department of Systems Medicine, Tor Vergata University, Rome, Italy; 8 Department of Neurosciences, Mental Health and Sensory Organs, Sapienza University, Rome, Italy; 9 Department of Neurosciences, Imaging and Clinical Sciences, University G D'Annunzio, Chieti-Pescara, Italy; 10 Demyelinating Disease Center Department of Neurology, San Salvatore Hospital, L'Aquila, Italy

**Keywords:** algorithms of treatment, lifestyle interventions, nabiximols, Smouldering Disease, spasticity-plus-syndrome

In the published article, an incorrect Funding statement was provided below:

“The authors declare no financial support was received for the research, authorship, and/or publication of this article. This project is the result of a collaboration between Protagon – healthcare communication agency – and the authors.”

The updated funding statement appears below:

“The authors declared that financial support was received for this work and/or its publication. This project is the result of a collaboration between Protagon—healthcare communication agency—and the authors. We thank Daniela Melisi and Marco Stacchiotti who provided editorial assistance, funded by Almirall S.p.A. The funder was not involved in the study design, collection, analysis, interpretation of data, the writing of this article, or the decision to submit it for publication.”

The original version of this article has been updated.

